# Smooth and large scale organometallic complex film by vapor-phase ligand exchange reaction†

**DOI:** 10.1039/d0ra00403k

**Published:** 2020-03-05

**Authors:** Myeonggeun Choe, Soyoung Kim, Hee Cheul Choi

**Affiliations:** Department of Chemistry, Pohang University of Science and Technology (POSTECH) 77 Cheongam-ro, Nam-gu Pohang-si 37673 Korea choihc@postech.edu

## Abstract

A simple and reliable method for the formation of smooth and large-scale organometallic complex thin films was developed. We applied chemical vapor deposition (CVD) for this. From the vapor-phase reaction of Mo(CO)_6_ and 2,2′-bipyridine, large-scale and highly smooth Mo(CO)_4_(2,2′-bipy) films were obtained. Regardless of the thickness, they show a high smoothness and stability in ambient conditions. Chemical structure and composition of the resulting film were confirmed by ^1^H-NMR, Raman, FT-IR spectroscopy and elemental analyses. Smooth and uniform surface of the resulting films was characterized using AFM. We believe that our method will provide great opportunities for the fundamental studies of traditional organometallic complexes and their applications by taking advantages of thin film geometry.

## Abstract 2

Highly-smooth Mo(CO)_4_(2,2′-bipy) thin film showing a color gradient depending on the thickness can be obtained by vapor-phase ligand exchange reaction.

## Graphical abstract

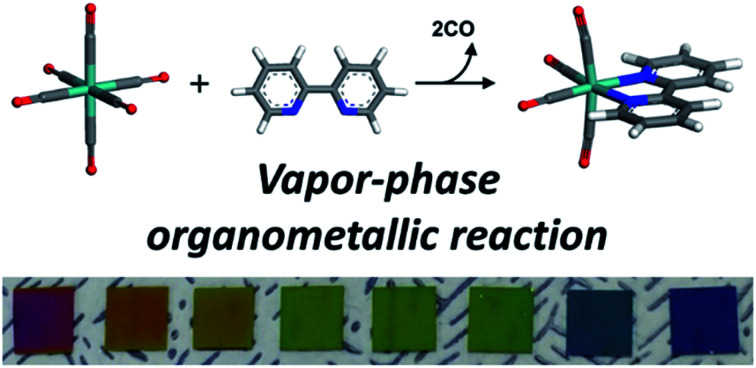

Fabrication of organometallic complexes (OMCs) into high-quality thin films would enable observation of previously-unseen chemical reactions, and allow elucidation of properties that are difficult to detect in other phases than thin films. In addition, the thin films would have various applications in fields such as electronics, photoelectronics, optics, photonics, magnets and spintronics.^1–6^ Most attempts to obtain high-quality OMC films have tried to coat pre-synthesized target complexes.^7^ However, these methods were mostly unsuccessful and if successful were not easily applied to general OMCs. Therefore, a new reliable method for the formation of high-quality OMC films is sought.

Direct formation of OMC films on a target substrate is a promising method because of their good reactivity and potentially interesting electrical and optical properties. OMCs have been synthesized in solution phase, but this method is more appropriate for obtaining three-dimensional structures than for films. Also, physical vapor deposition methods to pre-synthesize OMCs are also not appropriate due to their low vapor pressure and the possibility of decomposition. Hence, development of efficient synthesis methods to obtain large-scale and uniform OMC film remains a challenge.

Chemical vapor deposition (CVD) is an effective method to produce large-scale two-dimensional materials^8,9^ and to form films of polymers^10^ and metal–organic frameworks.^11^ Here, we report a rapid and highly efficient CVD method that uses *in situ* vapor-phase chemical reactions of precursors to synthesize highly-uniform thin films of OMCs. This method facilitates direct reaction of precursors without any disturbance by solvent or impurities, and therefore induces facile formation of pure, highly-uniform and smooth OMC films. It is useful for electrocatalytic CO_2_ reduction^12^ and ligand exchange reaction.^13^ We exploited a vapor-phase ligand exchange reaction between hexacarbonylmolybdenum(0) (Mo(CO)_6_) and 2,2′-bipyridine (2,2′-bipy). The reaction was conducted in a CVD system within 5 min to yield highly-uniform, smooth, and large-scale Mo(CO)_4_(2,2′-bipy) thin film for the first time. We then used the Mo(CO)_4_(2,2′-bipy) film in organometallic thin-film devices and measured their electrical properties.

High-quality OMC thin films were prepared using single-step CVD. The metal precursor was Mo(CO)_6_ and the ligand precursor was 2,2′-bipy. The goal was to obtain Mo(CO)_4_(2,2′-bipy) by a reaction that proceeds well in solution phase.^12,13^ Considering the vaporization temperature of Mo(CO)_6_ (94.45 °C) and 2,2′-bipy (113.75 °C) (Fig. S1†), we chose 123 °C as a target temperature for efficient and simultaneous evaporation of precursors. In the CVD system for vapor-phase organometallic reaction (Fig. 1a), Mo(CO)_6_ powder was placed 13.5 cm upstream from the center. And 2,2′-bipy powder was placed at the center. This arrangement exploits the temperature gradient in the furnace, and the higher vaporization temperature of 2,2′-bipy than of Mo(CO)_6_. A SiO_2_/Si target substrate was placed downstream from the center of the tube to collect product efficiently. The quartz tube was flushed using Ar, then the tube furnace was heated from room temperature to the target temperature at 10 °C min^−1^ (Fig. 1b). After 5 min of reaction at target temperature, the power to the furnace was turned off and the sample was allowed to cool passively to room temperature.

**Fig. 1:**
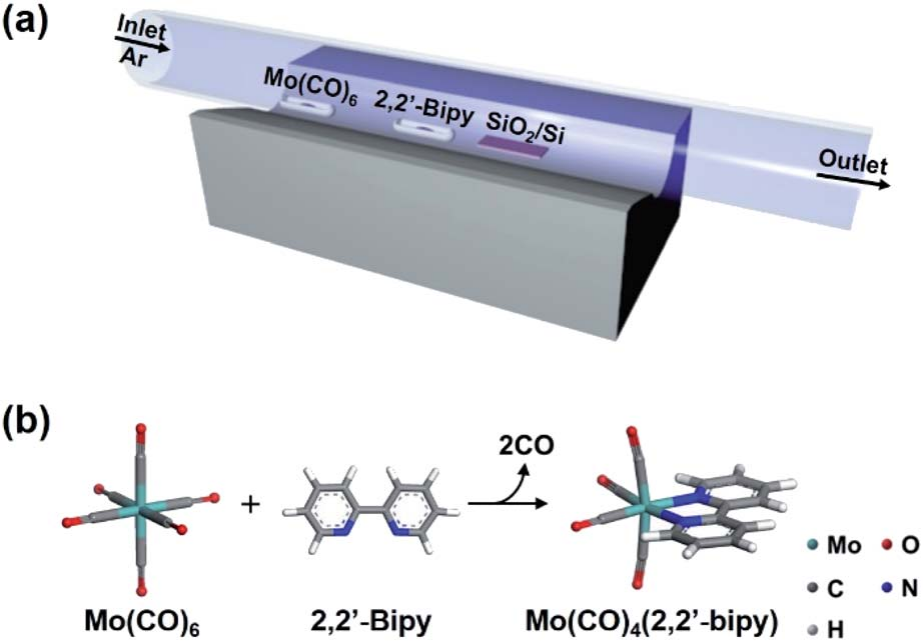
(a) Experimental scheme of the CVD system for the synthesis of Mo(CO)_4_(2,2′-bipy) thin film. (b) Molecular structure Mo(CO)_4_(2,2′-bipy) obtained by vapor-phase ligand exchange reaction between Mo(CO)_6_ and 2,2′-bipy.

The resulting large-scale Mo(CO)_4_(2,2′-bipy) film was highly uniform and had no notable physical defects or chunks (Fig. 2). We confirmed that the product did not undergo thermal decomposition at operating temperature (123 °C) (Fig. S2 and Table S1†). The surface of the resulting film was examined using a tapping-mode atomic force microscope (AFM). To measure the thickness of the resulting film, we etched away the film except for a part that was covered using Kapton tape as a shadow mask. The film was treated using CF_4_ at flow rate of 40 sccm and O_2_ plasma at 5 sccm with power of 150 W power, respectively. Etching time was determined depending on film thickness, from 30 to 90 min. After the process, the measured thickness was 38.6 nm (Fig. S3a†); the height distribution measured by AFM showed that the Mo(CO)_4_(2,2′-bipy) film was highly smooth and uniform surface (root mean square roughness *r*_RMS_ = 0.267 nm, Fig. 2b), which is similar to that of a bare SiO_2_/Si substrate (*r*_RMS_ = 0.249 nm, Fig. S3b†). A scanning electron microscopy (SEM) image (Fig. S4†) of the obtained film shows uniform and homogenous surfaces over a large area. Energy-dispersive electron energy loss spectroscopy (EELS) analysis confirmed the presence of Mo, C, N, and O (Fig. S5†). These results demonstrate that CVD is a highly efficient approach to obtain highly-uniform OMC films.

**Fig. 2:**
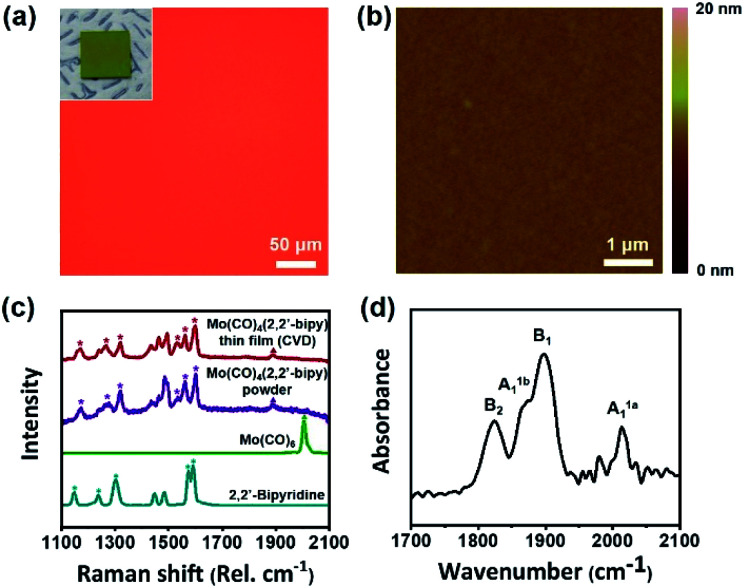
(a) Optical microscopy image, photograph (inset), and (b) AFM image of the obtained Mo(CO)_4_(2,2′-bipy) thin film. (c) Raman spectra of the Mo(CO)_4_(2,2′-bipy) powder (purple), and two precursors (Mo(CO)_6_ (green), 2,2′-bipy (coral blue)). (d) FT-IR spectrum of Mo(CO)_4_(2,2′-bipy) thin film showing four carbonyl stretching bands.

The chemical structure of the resulting film was confirmed by Raman and FT-IR analyses (Fig. 2d). For a direct comparison of the chemical structure of the resulting film with a reference, we separately synthesized Mo(CO)_4_(2,2′-bipy) bulk powder by using a microwave-assisted synthesis method described elsewhere^14^ (ESI†). The obtained powder was red (Fig. S6†) and its structure as confirmed by ^1^H-NMR was identical to that reported in the reference paper.^14^ Raman spectra (Fig. 2c) were obtained from precursors, product film, and reference powder. The carbonyl stretching band was observed at 1900–2000 cm^−1^, and several bipyridine stretching bands were observed at 1100–1600 cm^−1^.^15^ The stretching bands of the aromatic rings of 2,2′-bipy (asterisks) and the carbonyl stretching bands (triangle) were clearly resolved. The vibrational band of Mo(CO)_4_(2,2′-bipy) film (red) is almost identical with the reference Mo(CO)_4_(2,2′-bipy) powder (purple).

To identify chemical species observed in Raman spectra, we conducted density functional theory (DFT) calculations using Dmol^3^ modules in Material Studio program packages (details in ESI†). The six representative bands observed at 1170.8, 1263.5, 1322.0, 1535.5, 1563.9 and 1600.1 cm^−1^ in the Raman spectrum of Mo(CO)_4_(2,2′-bipy) film were confirmed as molybdenum-coordinated bipyridine ligand stretching bands (red), which are shifted and split from the original stretching bands of bipyridine precursor (1147.6, 1236.4, 1303.4, 1574.1, and 1590.2 cm^−1^, blue). Also, the carbonyl stretching band shifted from 2005.9 cm^−1^ to 1888.2 cm^−1^ because to the new coordination bonds between molybdenum and 2,2′-bipy ligand has a weaker π-accepting ability than the previously-bonded carbonyl ligands.^16^ The DFT calculations generated Raman vibrational modes of precursors and film (ESI Videos†). Also, we measured the FT-IR spectrum of Mo(CO)_4_(2,2′-bipy) film by using an attenuated total reflection (ATR) mode (650–4000 cm^−1^). The spectrum (Fig. 2d) shows carbonyl stretching bands at 2014, 1898, 1866, and 1824 cm^−1^, which correspond respectively to A^1a^_1_, B_1_, A^1b^_1_, and B_2_ vibrational modes of carbonyl ligands interacting to the six-coordinated molybdenum complex; this result is a good match to reference data.^17^

For further characterization, we measured ^1^H-NMR of dissolved Mo(CO)_4_(2,2′-bipy) film in CDCl_3_. The NMR peaks (Fig. S7,† triangles) match well with the reference NMR peaks of Mo(CO)_4_(2,2′-bipy) powder,^14^ and also confirmed the presence of small amount of 2,2′-bipy precursor (squares). Grazing-incidence wide-angle X-ray scattering (GI-WAXS) measurements (Fig. S8†) we confirmed the amorphous structure of the resulting film.

One of the big advantages of CVD is that the thickness of the resulting film can be controlled easily by changing the amount of precursors. Mo(CO)_4_(2,2′-bipy) films were formed on SiO_2_/Si substrate by using different amounts of precursors; the films showed a continuous color gradient that depended on the thickness (Fig. 3a). The thickness can be controlled from the range of tens of nanometers to micron scale; all surfaces were highly smooth (Fig. S9†).

**Fig. 3:**
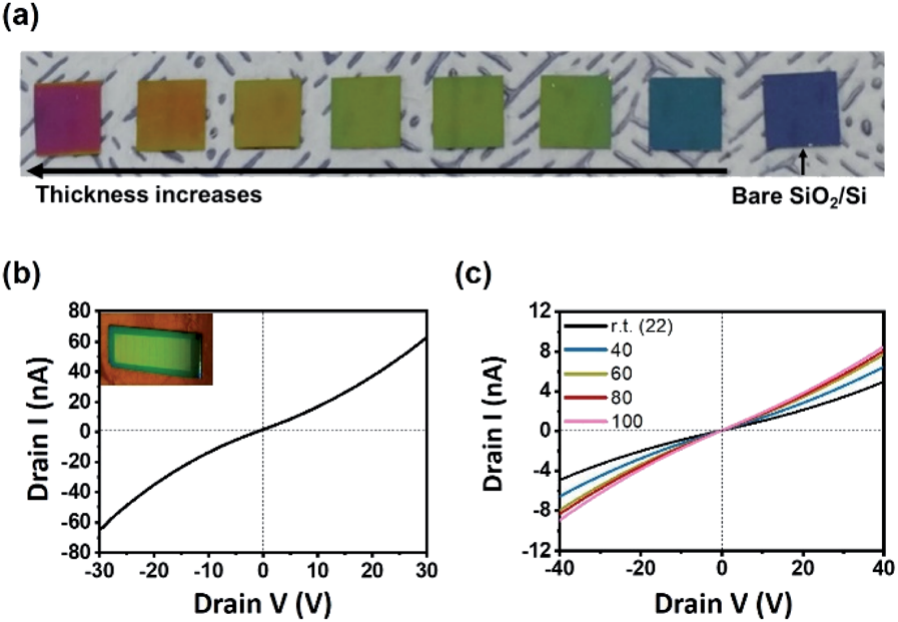
(a) Photograph of Mo(CO)_4_(2,2′-bipy) films showing a continuous color gradient depending on the thickness. The rightmost sample is a bare SiO_2_/Si substrate. (b) *I*–*V* characteristic curve of Mo(CO)_4_(2,2′-bipy) thin film; inset: photograph of the fabricated device. (c) *I*–*V* characteristics at rt ≤ *T* ≤ 100 °C for DC voltage −40 to 40 V.

To exploit the geometrical advantage of the thin film for device fabrication, field-effect transistor (FET) electronic devices with a channel length of 20 mm (Fig. 3b, inset) were fabricated (ESI and Fig. S10†). The *I*_ds_–*V*_ds_ curve (Fig. 3b) of the resulting device indicated that the highest electrical conductance was 1.90 × 10^−9^ S, and the highest conductivity was 1.99 × 10^−5^ S m^−1^. The linear characteristic of *I*–*V* curve is a sign of ohmic contact between film and electrode, as a consequence of the uniform and smooth surface of Mo(CO)_4_(2,2′-bipy) film. The electrical conductance of Mo(CO)_4_(2,2′-bipy) film increased as the temperature was increased from room temperature to 100 °C (Fig. 3c); this result shows the semiconducting nature of the Mo(CO)_4_(2,2′-bipy) film. To compare the electrical property of film with reference powder, Mo(CO)_4_(2,2′-bipy) powder was pelletized and fabricated on a SiO_2_/Si substrate. The pellet was rough and thick, so we used silver paste as an adhesive electrode. The *I*–*V* characteristic curve (Fig. S11†) of Mo(CO)_4_(2,2′-bipy) pellet exhibits non-ohmic current–voltage characteristics between the pellet and the electrode, and eventually failed to measure the electrical property of the complex. These results demonstrate that the intrinsic properties of organometallic materials requires synthesis of uniform and smooth organometallic film.^18,19^

In summary, we synthesized large-scale, highly-uniform, smooth, and thickness-controllable Mo(CO)_4_(2,2′-bipy) thin films by vapor-phase ligand exchange reaction that exploits chemical vapor deposition (CVD). Our strategy facilitates the vapor-phase reaction of precursors without any disturbance of solvent or impurities, and also yields a suitable smooth film geometry that is advantageous for various electrical and optical device applications. FET devices that use Mo(CO)_4_(2,2′-bipy) thin film exhibit semiconducting behaviour. We believe that these results provide insights that will guide development of novel strategies to synthesize various OMC films for use in various electrical and optical applications.

## Conflicts of interest

There are no conflicts to declare.

## Supplementary Material

RA-010-D0RA00403K-s001

RA-010-D0RA00403K-s002

RA-010-D0RA00403K-s003

RA-010-D0RA00403K-s004

RA-010-D0RA00403K-s005
