# Supplementary material for: Smooth and large scale organometallic complex film by vapor-phase ligand exchange reaction
Source: RSC Adv. 2020 Mar 5;10(16):9536–8. doi: 10.1039/d0ra00403k (PMC9050125; doi:10.1039/d0ra00403k)
Supplement: RA-010-D0RA00403K-s001 [file RA-010-D0RA00403K-s001.pdf]

Electronic Supplementary Material (ESI) for RSC Advances.
This journal is © The Royal Society of Chemistry 2020

Supplemental Information

Smooth and Large scale Organometallic Complex Film by Vapor-Phase Ligand Exchange Reaction

Ms. Myeonggeun Choe, Dr. Soyoung Kim, and Prof. Hee Cheul Choi

[a] Department of Chemistry, Pohang University of Science and Technology (POSTECH), 77 Cheongam-ro, Nam-gu, Pohang-si, 37673, Korea

* Corresponding author: H. C. Choi, E-mail: choihc@postech.edu

Experimental Procedures

1: General experimental information

$Mo(CO)_4$(2,2'-bipy) powder and film were synthesized from commercially available hexacarbonylmolybdenum(0) ($Mo(CO)_6$, strem chemicals, INC.) and 2,2'-bipyridine (2,2'-bipy, >99.0 % (T), Tokyo chemical industry co., LTD.) without further purification. Thermogravimetric analysis (TGA) spectra were obtained to confirm the vaporization temperature of precursors ($Mo(CO)_6$ and 2,2'-bipyridine) using a thermogravimetric analyzer (SCINCO model no.1000).

2: Syntheses

Synthesis of $Mo(CO)_4$(2,2'-bipy) thin film by CVD method

Precursors were located in ceramic crucibles at equal mass ratio. The amount of precursors were controlled from 3 to 8 mg for the thickness control of $Mo(CO)_4$(2,2'-bipy) film. the crucibles containing $Mo(CO)_6$, precursor and 2,2'-bipy were located at the center and 13.5 cm upstream from the center of a protecting quartz tube, respectively. A SiO_2/Si (100) substrate was also located at 7 cm downstream from the center of the tube. We flushed argon gas to purge the quartz tube for 5 minutes at 600 sccm. After purging, we increased furnace temperature up to 123 ^oC with Ar flowing at 600 sccm and maintained at target temperature for 5 minutes. After reaction, we cooled down the furnace temperature naturally by turning the power off to room temperature.

Microwave synthesis of $Mo(CO)_4$(2,2'-bipy) powder

$Mo(CO)_4$(2,2'-bipy) reference powder was synthesized using a multimode microwave oven (MARS®-5 CEM Corp.). After adding precursor solution prepared by dissolving 0.65 g of 2,2'bipyridine and 1.00 g of $Mo(CO)_6$ in toluene to the single XpressTeflon vessel (CEM corp.), we reacted the tube for 30 seconds at 453 K in the microwave oven. After reaction, the precipitates were washed using *n*-hexane. Finally, we obtained a brick-red solids.[1] (Fig S5)

3: Structural and spectroscopic characterization

^{1}H-NMR spectra were measured using a Bruker Advance III (Ultrashield Plus 500 MHz magnet) spectrometer. The NMR peaks appeared at following positions: δ 9.161, δ 8.690, δ 8.401, δ 8.116, δ 7.936, δ 7.827, δ 7.395, δ 7.318 (Fig S6). Among them, δ 9.161(*d*, 2H, H5 and H14), δ 8.116 (*d*, 2H, H8 and H11), δ 7.936 (*t*, 2H, H7 and H12), and δ 7.395 (*t*, 2H, H6 and H13) are identical with reference NMR peaks of Mo(CO)4(2,2'-bipy) bulk powder[1] and others peak: δ 8.690 (*d*, 2H, H4 and H4'), δ 8.401 (*d*, 2H, H1 and H1'), δ 7.827 (*t*, 2H, H2 and H2') and δ 7.318 (*m*, 2H, H3 and H3') are responsible for the unreacted small amount of 2,2'-bipy precursor. (Fig S6) CHN microanalysis of the $Mo(CO)_4$(2,2'-bipy) was performed using an elemental analyzer (Vario MICRO, ANALYSENSYSTEME GMBH). The results of **1** (calcd. (%) C-44.24; H-1.830; N-7.47) and **2** (calcd.(%) C-44.41; H-1.738; N-7.54) indicate that the $Mo(CO)_4$(2,2'-bipy) does not undergo thermal decomposition at operating temperature (123 °C) for operating time (5min). (**1**: unheated $Mo(CO)_4$(2,2'-bipy), **2**: $Mo(CO)_4$(2,2'-bipy) heated at 123 °C for 10min) Raman spectra were obtained using a WITECH Alpha 300 R Raman spectroscope equipped with a 532 nm laser. Fourier-transform infrared (FT-IR) spectra were recorded on a Varian 670-IR FT-IR spectrometer by attenuated total reflection (ATR) mode (650-4000 cm^{-1}) and sample was grown on a gold deposited SiO_2/Si substrate. The surface morphologies of the resulting thin films were characterized by tapping mode atomic force microscope (AFM, Nanoscope IIIa machine, Digital Instrument Inc.) and AFM images with height profiles were treated using Nanoscope IIIa (Digital Instrument Inc.) program. After the platinum coating on the resulting film to prevent electron charging, the surface morphology of the thin film was also characterized by SEM (JSM-7410F, JEOL). The uniformity of the resulting film was confirmed by electron energy loss spectroscopy (EELS) by HR-FE-TEM-(2200FS with Cs-corrected TEM) (JEOL JEM-2200FS (with Image Cs-corrector), and the samples for EELS measurement were directly grown on a TEM grid (200 mesh Cu, Ted Pella Inc.) by CVD method. The crystallinity of the resulting film was confirmed through WAXS (Wide-angle X-ray scattering) measurement in 3C beamline at Pohang Accelerator Laboratory (PAL).

4: Device fabrication and measurement

$Mo(CO)_4$(2,2'-bipy) thin film field-effect transistor type electronic devices were fabricated using a copper wire with diameter of 30 μm as a shadow mask. After attaching wire on the film, we covered the edge of the substrate once again to fix the copper wires using carbon tape. After that, titanium and gold were deposited 5nm and 20~50nm thick (depending on film thickness), respectively. Detailed experimental procedure is described in Fig S8.

All current measurements were conducted using a probe station (M6VC, MS TECH) and a semiconductor analyzer (Keithley, 2600) at room temperature except for the temperature dependent *I-V* characteristics experiments (Fig 3c). Temperature during the measurement was controlled by a hot chuck controller (MST-1000H, MS TECH).

5: Density functional theory (DFT) calculations.

To analyze frequency properties of precursor and product, respectively, we did DFT calculation using $Dmol^3$ modules in Material Studio program packages. The project was conducted using local PWC function setup.

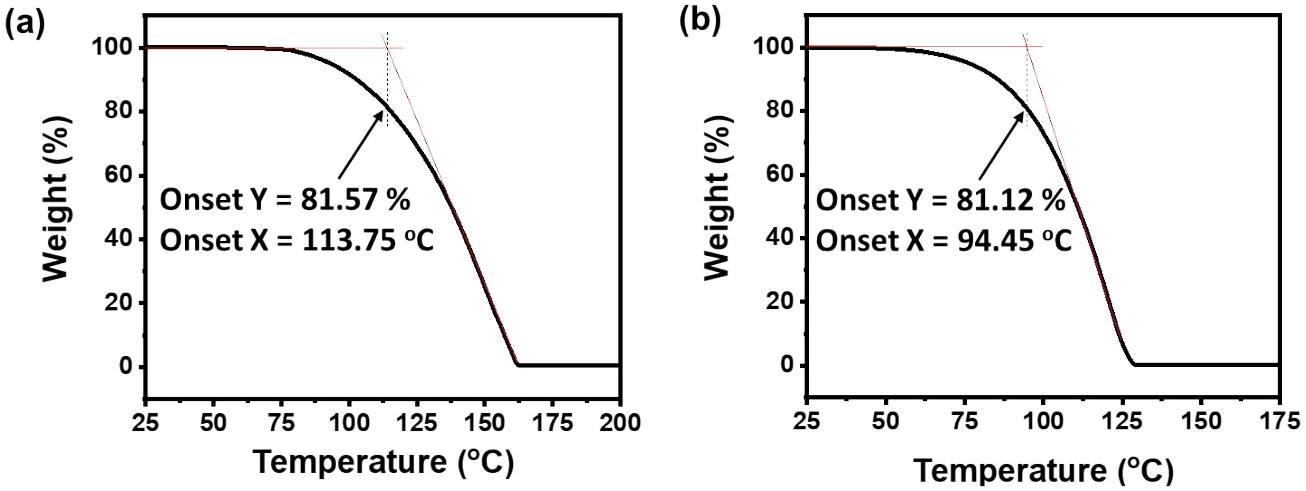

Fig S1. TGA results of (a) 2,2′-bipy that shows onset temperature of 113.75 °C and (b) $Mo(CO)_6$ that shows onset temperature of 94.45 °C.

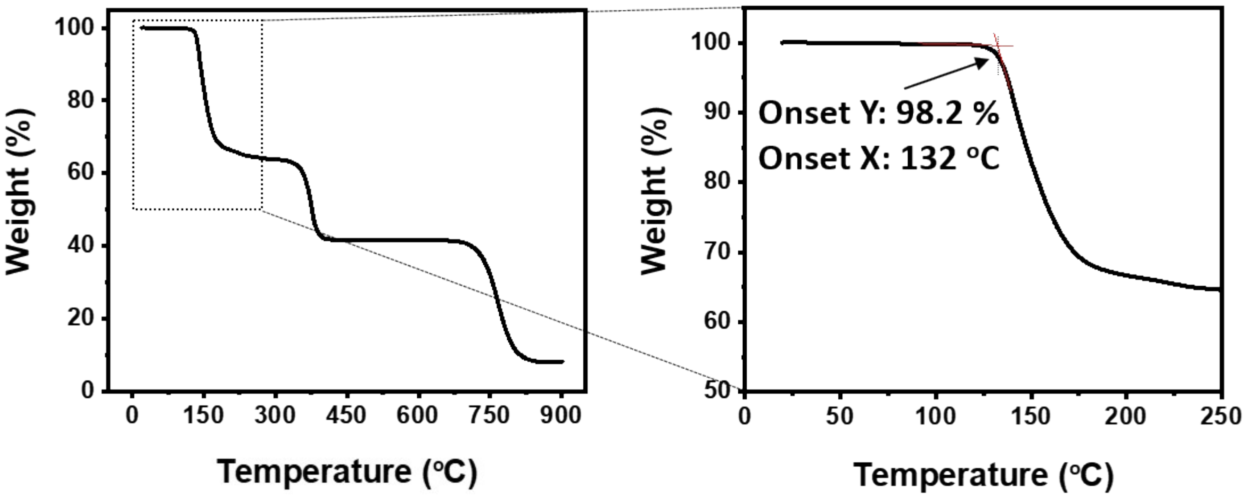

Fig S2. TGA results of $Mo(CO)_4$(2,2'-bipy) at Ar. The arrow point to the first onset of weight loss and the first weight loss occurred at 132 °C.

Sample	N [%]	C [%]	H [%]
**1**	7.54	44.41	1.738
**2**	7.58	44.43	1.774

Table S1. CHN microanalysis data of $Mo(CO)_4$(2,2'-bipy).

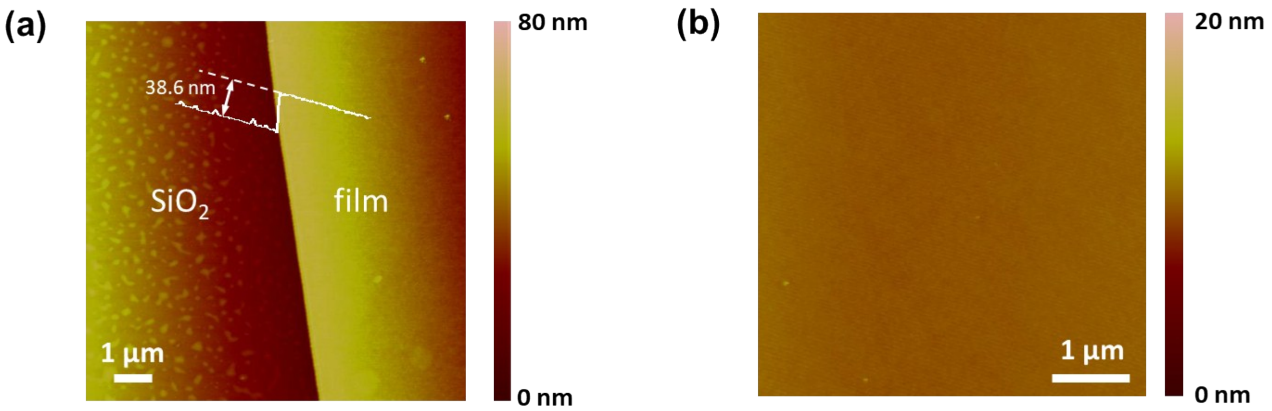

Fig S3. (a) AFM image with height profile of the product film used in device fabrication. (b) AFM image of a bare SiO_2/Si substrate having $r_{RMS, SiO2/Si}$ of 0.249 nm.

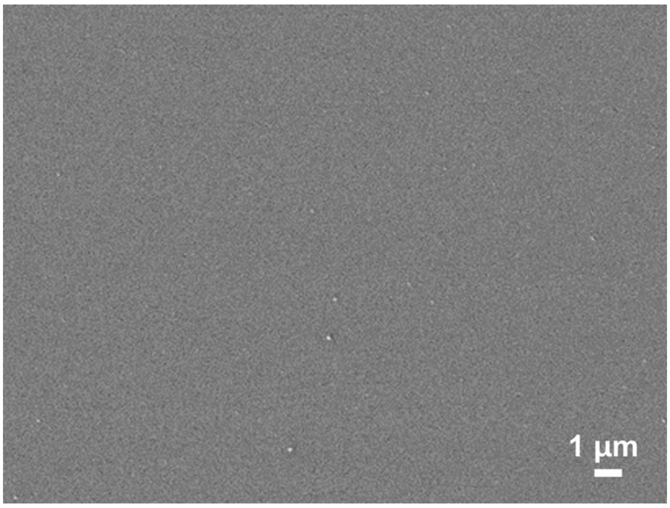

Fig S4. The SEM image of obtained thin film having uniform and smooth surface.

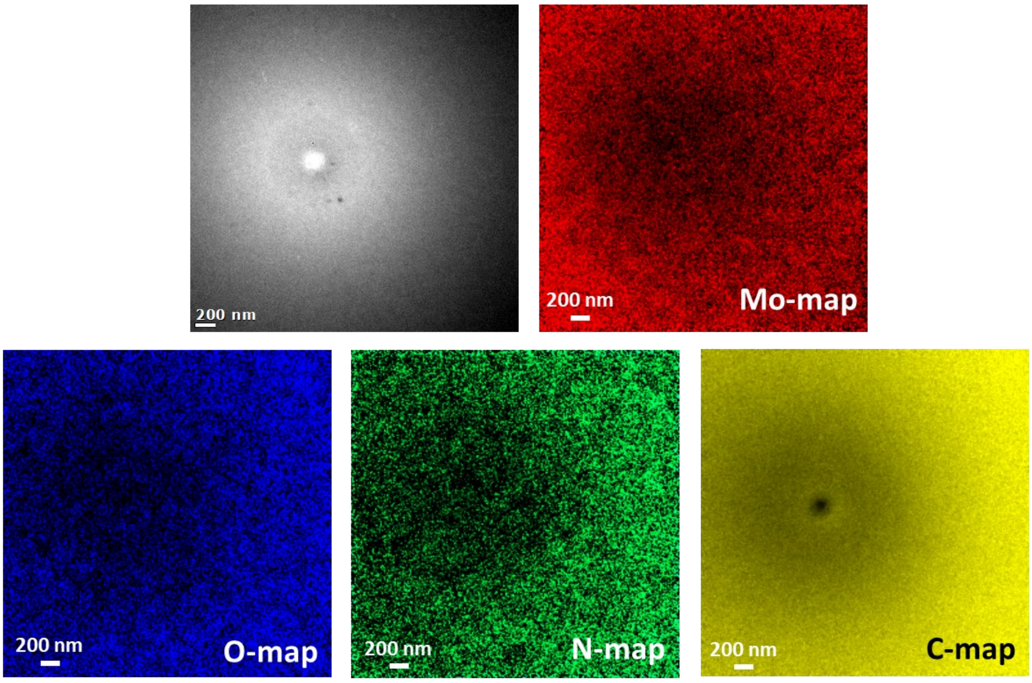

Fig S5. EELS mapping images of M-edge of molybdenum oxygen and K-edges of oxygen, nitrogen, and carbon of obtained $Mo(CO)_4$(2,2'-bipy) thin film.

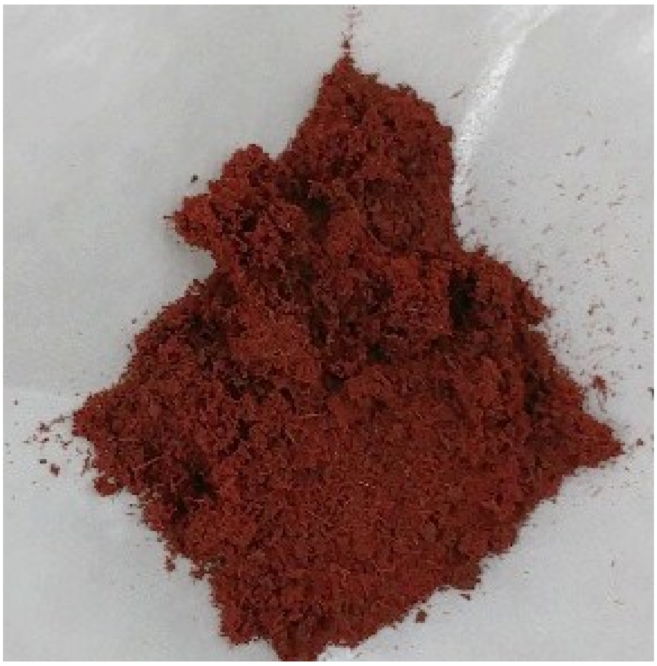

Fig S6. Photograph of synthesized $Mo(CO)_4$(2,2'-bipy) reference powder.

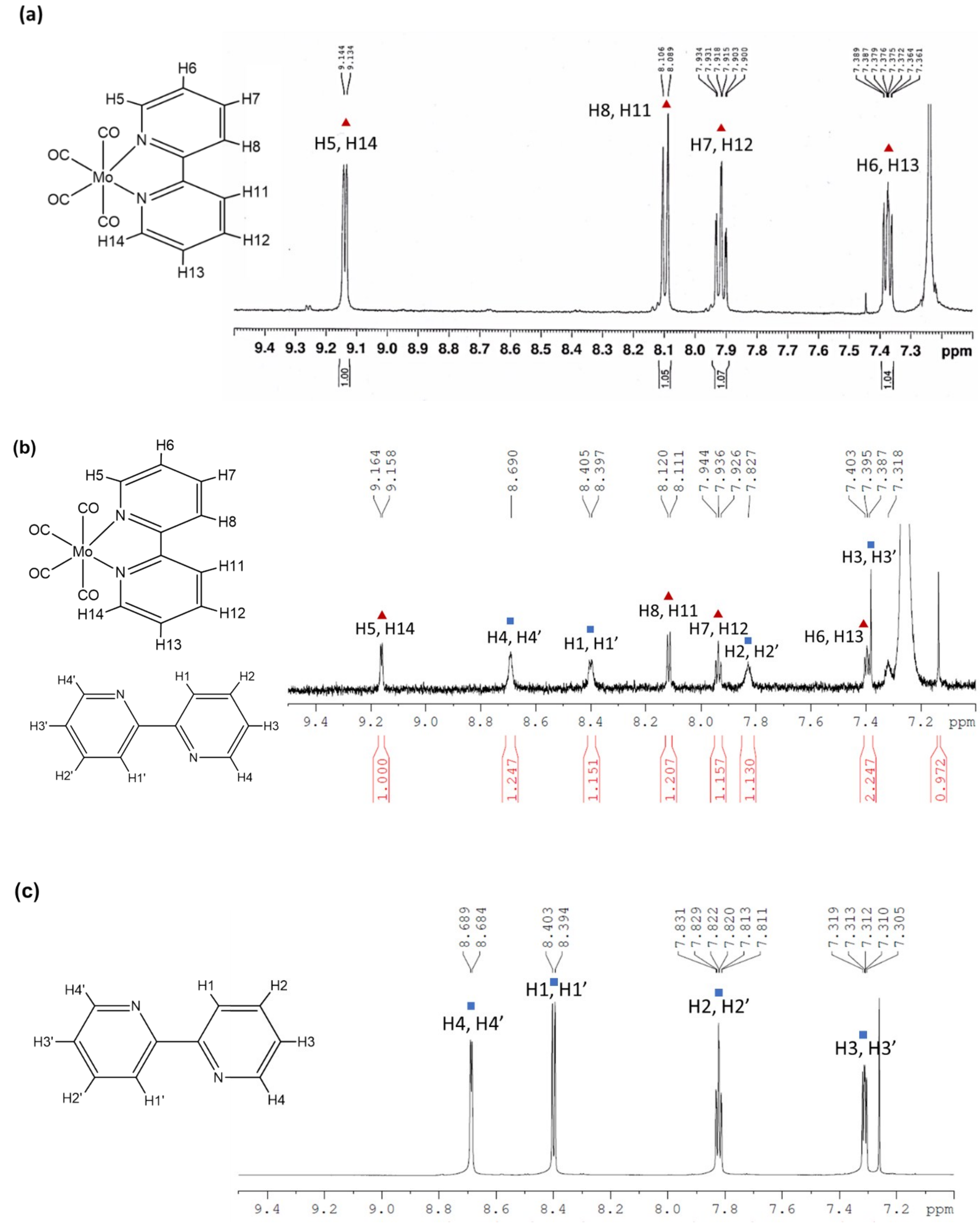

Fig S7. ^{1}H-NMR spectra of (a) $Mo(CO)_4$(2,2'-bipy) reference powder, (b) $Mo(CO)_4$(2,2'-bipy) thin film grown by CVD, (c) 2,2'-bipy precursor. All samples were prepared using $CDCl_3$.

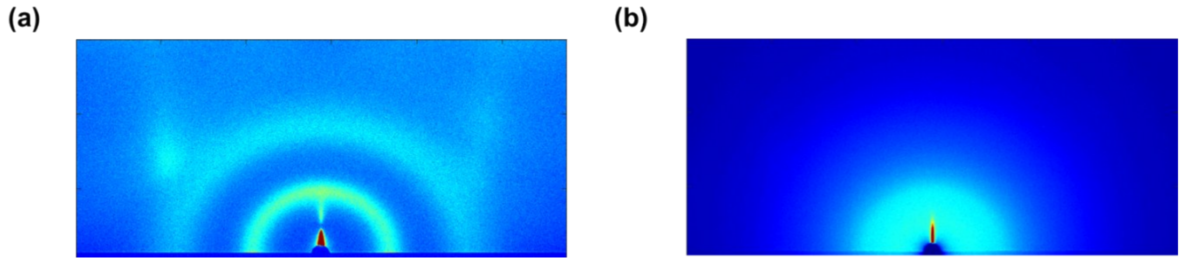

Fig S8. Two-dimensional GI-WAXS images (a) in vacuum, and (b) in ambient condition of $Mo(CO)_4$(2,2'-bipy) thin film.

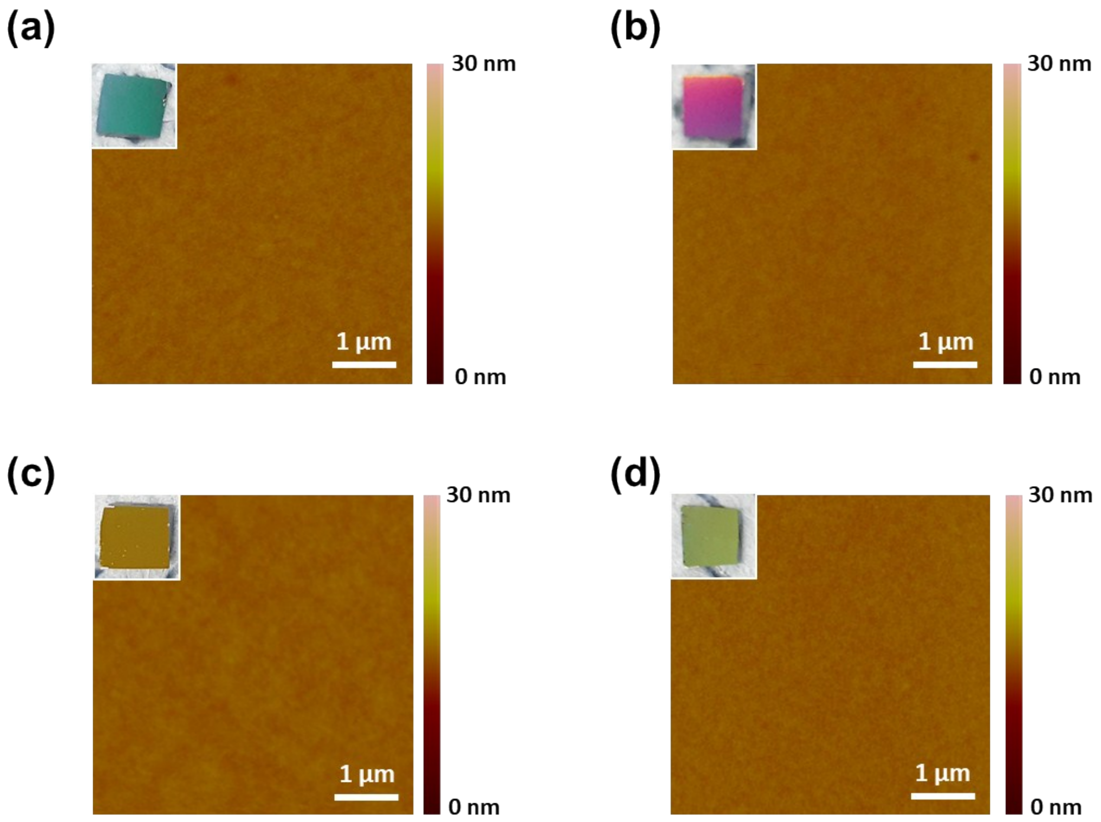

Fig S9. AFM images with height scale bars and photographs (inset) of the samples having various film thicknesses. (a) and (b) shows micron scale, (c) and (d) shows tens of nanometer scale film. The root mean square roughness ($r_{RMS,Mo(CO)4(2,2'\text{-}bipy)}$) were 0.296, 0.308, 0.286, 0.308 nm, respectively, which means each film is highly-smooth regardless of the thickness.

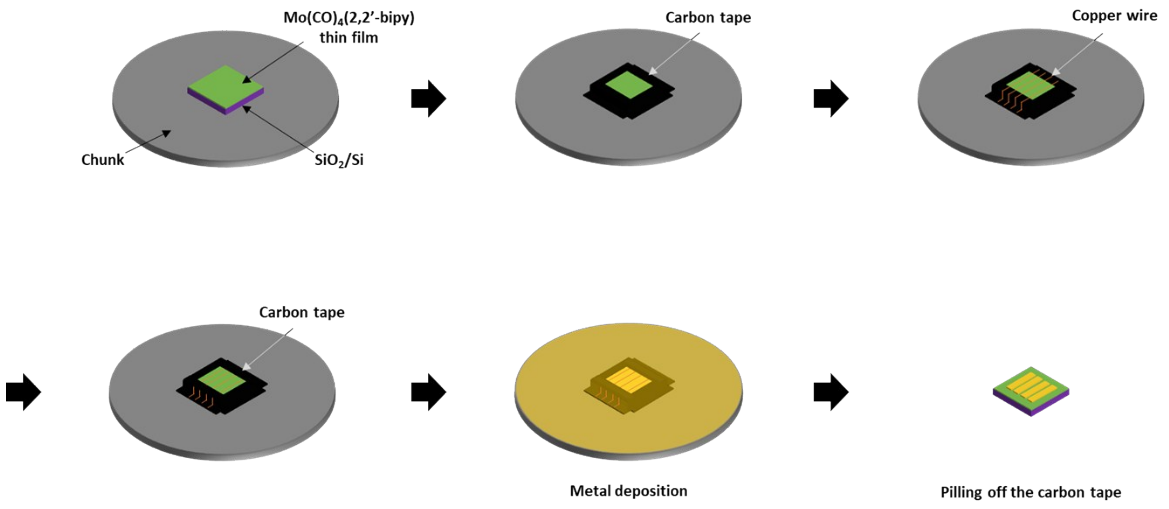

Fig S10. Experimental scheme for device fabrication of $Mo(CO)_4$(2,2’-bipy) thin film.

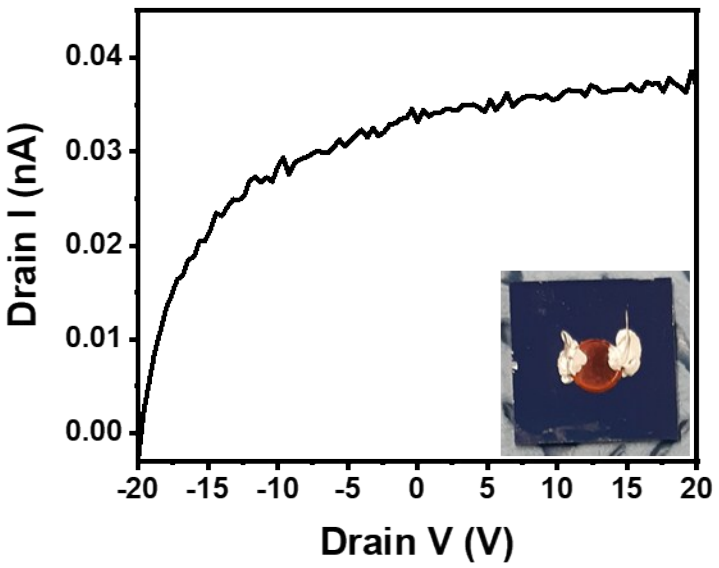

Fig S11. *I-V* characteristic curve of $Mo(CO)_4$(2,2'-bipy) pellet; inset: photograph of pellet device.

References

1 S. S. Braga, A. C. Coelho, I. S. Gonçalves and F. A. Almeida Paz, *Acta Crystallogr. Sect. E Struct. Reports Online*, 2007, **63**, m780–m782.
